# Modelling the impact of environmental and social determinants on mental health using generative agents

**DOI:** 10.1038/s41746-024-01422-z

**Published:** 2025-01-17

**Authors:** Joseph Kambeitz, Andreas Meyer-Lindenberg

**Affiliations:** 1 Department of Psychiatry and Psychotherapy, Faculty of Medicine and University Hospital, University of Cologne, Cologne, Germany; 2 Department of Psychiatry and Psychotherapy, Central Institute of Mental Health, Medical Faculty Mannheim, University of Heidelberg, 68159 Mannheim, Germany; 3 German Centre for Mental Health (DZPG), Partner Site Heidelberg/Mannheim/Ulm, Mannheim, Germany

**Keywords:** Human behaviour, Risk factors, Psychology and behaviour, Psychiatric disorders, Computational science

## Abstract

Mental health is shaped by socio-environmental determinants, yet traditional research approaches struggle to capture their complex interactions. This review explores the potential of generative agents, powered by large language models, to simulate human-like behaviour in virtual environments for mental health research. We outline potential applications including the modelling of adverse life events, urbanicity, climate change, discuss potential challenges and describe how generative agents could transform mental health research.

## Social and environmental determinants of mental health

Environmental factors refer to external conditions and stimuli in the physical surroundings - such as pollution, climate patterns, noise, and urban infrastructure - many of which can affect mental health. Social factors encompass the features of interpersonal relationships, community structures, social networks, and cultural norms that shape individual well-being and influence mental health outcomes. Both social and environmental factors play an essential role for the development and progression of mental disorders. Conditions such as affective disorders, psychotic disorders, anxiety disorders, personality disorders, dementia or substance use disorders have all been linked to socio-environmental influences^1–5^. For example, several key aspects of urban living - such as social deprivation, high population density, limited access to green spaces or environmental pollution - have been identified as significant risk factors for the development of conditions like psychotic disorders^6–9^ or depression and anxiety^10^. Beyond contributing to the development of mental disorders, social and environmental factors have significant impact on their trajectory once they manifest. For example, social contacts, employment, adverse life events, education and access to healthcare have been linked to favourable outcomes across different mental disorders^11,12^. Importantly, many of these socio-environmental risk factors are considered to be modifiable, offering significant opportunities for interventions to improve mental health outcomes^13^. Moreover, social and environmental factors often interact in complex ways, as illustrated by the concept of resilience. Resilience implies that the adverse effects of environmental factors can be partially mitigated by individual traits, such as optimism^14^, protective social influences, such as the support provided by family members or strong social networks^15^ or by environmental factors such as green space^16^. These interactions highlight the importance of considering both social and environmental dimensions together when assessing mental health risks and outcomes. As urban environments continue to expand and encompass the majority of the world’s population^17^, understanding the complex interplay of social and environmental factors and mental health becomes increasingly critical for public health strategies and interventions.

## Challenges in research of social and environmental determinants of mental health

Current research on the social and environmental determinants of mental health predominantly relies on observational data. Several methodologies have been employed to infer causality in this context (e.g. structural equation modelling, propensity score matching, Mendelian randomization or Bayesian networks)^18^. However, these methods often rely on assumptions that may not hold in real-world settings or are difficult to verify, thereby limiting their effectiveness in identifying causal pathways. Moreover, most research focuses on *individual* social or environmental factors, or aggregates them into summary measures such as an exposome score^19^ or indices of social vulnerability ^20^, overlooking the complex and multifaceted interactions between these influences^21^. If such interactions are investigated systematically they can be found to be highly superadditive^22^.

A complementary approach to investigate socio-environmental influences involves using “agents”^23^. Agents are typically computational entities that interact autonomously within virtual environments. They are programmed to simulate human behaviour by interacting with the environment or other agents, or by making decisions based on past experiences, memory, and internal rules. One key advantage of agent-based approaches is that they are able to generate emergent phenomena which are not explained by a system’s individual parts^23^. Some early works have employed agent-based approaches to model health-related behaviours^24–31^. However, for studying *mental* health current agent-based approaches face significant limitations. First, these simulations are typically based on highly simplified environments, resulting in limited ecological validity. Furthermore, these simulations often prioritize observable agent behaviors as their primary outcomes. While behavior is an important aspect of mental health, this focus neglects the subjective experiences and internal states that are central to understanding mental health. To better capture the complexity of mental health outcomes, it is necessary to integrate detailed readouts related to psychopathological symptoms, such as mood, anxiety, or stress. Without this level of granularity, the simulations fail to reflect the nuanced and multifaceted nature of mental health and its determinants.

Given these challenges, there is an urgent need for advanced research methodologies that can robustly identify causal relationships among socio-environmental determinants and ultimately allow for more effective mental health interventions.

## Large-language models and generative agents for modelling human behaviour

A recent line of research employed large-language models (LLMs) to create *generative agents* with the aim to simulate human behaviour^32,33^. Interestingly, this approach has the potential to overcome some of the aforementioned limitations of previous simulation endeavours including aspects of the limited ecological validity and the unspecific read-outs.

In general, LLMs are a subset of artificial intelligence models designed to process and generate data in textual formats, demonstrating remarkable performance across a wide range of tasks. Models like GPT-4, LLaMA, and Mistral are prominent examples, trained on vast corpora of text as well as image or video data that encapsulate diverse aspects of human experience and knowledge. Given that the training data used to develop these models is predominantly generated by humans, reflecting their thoughts, emotions, and behaviours, it is unsurprising that LLMs have shown a capacity to recreate plausible human behaviour in specific contexts^34–36^. For example, traumatic narratives make LLMs score higher on anxiety questionnaires^37^. Moreover, inducing “anxiety” in LLMs alters their responses in cognitive tasks^38^ indicating that LLMs can mimic human cognitive processes and thus offer a novel tool for investigating thought and behaviour^39^.

Recent studies have also explored the potential applications of LLMs in medicine, identifying several promising uses, particularly in psychiatry^40^. For instance, LLMs show good performance in medical question answering in medical exams and case reports^41^, can perform diagnostic interviews^42^ and contain information relevant to mental disorders^43^.

**Fig. 1:**
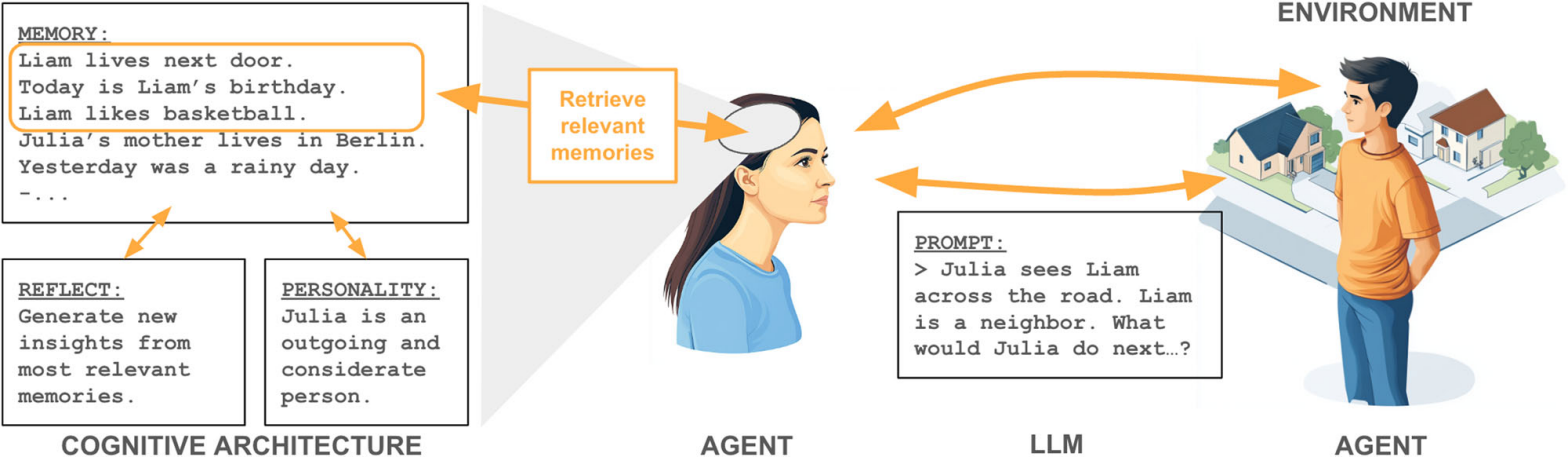
Generative agent framework for simulating human behavior. Generative agents utilise prompts to LLMs to integrate environmental cues with stored memories, enabling the generation of contextually appropriate actions^32^. A retrieval mechanism ranks memories based on criteria such as situational relevance, selecting those most pertinent for inclusion in the current prompt to the LLM. Personality traits and other individual characteristics can be incorporated into the prompt to ensure consistent, character-driven behaviour. The ability to reflect allows generative agents to generate new insights from past experiences and to store them as new memories.

Recent work has explored the integration of LLMs into generative agents to simulate human behavior, with a notable study by Park et al. demonstrating significant progress in this domain^32^. In their work, generative agents were employed in a virtual village of houses and roads in which the agents could interact autonomously, generating behaviors and social interactions that resemble human dynamics. The key innovation in Park et al.’s approach lies in the incorporation of a cognitive architecture that allows for a much richer repertoire of behaviors and interactions, enabled by modern LLMs (Fig. 1). This architecture includes a long-term memory system that stores past experiences, which are then retrieved based on factors like recency, importance, and relevance to the current context. When confronted with a novel situation, the agents query an LLM while incorporating these relevant memories into the prompt, ensuring that their responses are contextually grounded. Additionally, these agents possess the ability to reflect - an iterative process where they review past experiences, draw insights, and adapt their behaviors accordingly. This reflective mechanism enhances the realism of their interactions and decision-making, enabling them to exhibit character-consistent behaviour. By combining these features, Park et al.’s generative agents go beyond the capabilities of traditional simulation approaches. They can generate nuanced social interactions, exhibit adaptive behaviors, and capture a broader spectrum of human experiences, making them a powerful tool for investigating the intricate interplay between social and environmental factors in mental health research.

## Research applications of generative agents for investigating socio-environmental determinants of mental health

**Fig. 2:**
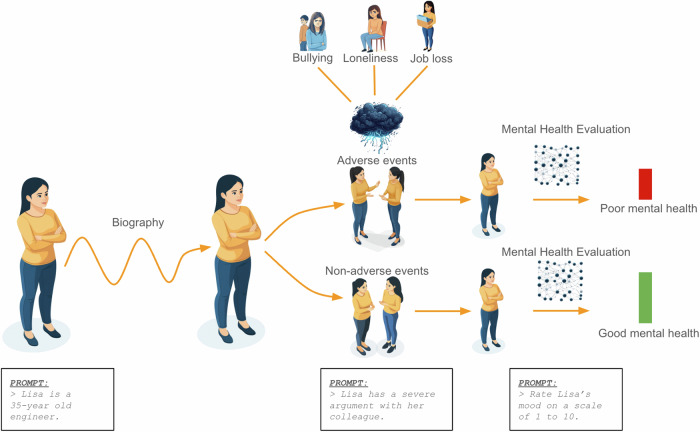
Using LLM-based generative agents to investigate the effects of adverse life events on mental health. Generative agents can be created with personalized biographies and exposed to virtual adverse and non-adverse life events. Adverse events, such as job loss, bullying, or loneliness can simulate stressors with negative impact on mental health. Subsequently agents can be prompted to self-report their mood or mental health status using standardized scales enabling the assessment of their mental health in these scenarios.

Generative agents could be embedded within virtual environments, enabling simulations of socio-environmental systems to study mental health. These environments could include data-driven replications of actual urban settings, allowing for the examination of specific geographic and structural factors (e.g., population density, proximity to green spaces, and access to mental health services) and their impacts on mental health outcomes. Within these simulations, agents could freely interact with each other and their surroundings, offering the opportunity to systematically manipulate social and environmental variables (Fig. 2). These virtual environments would allow for the alteration of agents’ biographical backgrounds, personality traits, or cognitive characteristics (e.g., long-term memory). Due to the dynamic nature of such simulations, continuous processes with relevance to mental health such as ageing or migration could be modelled efficiently. As the agents are based on LLMs, they could easily be prompted with established mental health questionnaires, enabling them to self-report symptoms (Fig. 2)^37^. Alternatively, generative agents could be programmed to function as virtual psychologists, capable of detecting symptoms and diagnosing disorders based on interactions with other agents. This generative agent framework could offer a novel method for experimentally investigating how socio-environmental factors influence mental health outcomes, potentially advancing our understanding of mental health in real-world contexts.

### Microlevel simulations of socio-environmental systems

Microlevel environments, such as families, dyads (pairs of individuals), or peer groups, are shaped significantly by individual characteristics (e.g., personality traits, beliefs, values, cognitive attributes) and the nature of their interpersonal relationships (e.g., dyadic interactions, social networks, group dynamics). Socio-environmental factors within these systems, such as childhood trauma^44–46^, bullying^47,48^, and loneliness^49,50^, have been shown to exert profoundly detrimental effects on mental health (Table 1). While epidemiological and qualitative research has provided valuable insights into these phenomena, agent-based simulations offer a complementary framework that enables the exploration of potentially causal mechanisms and dynamic interactions that are challenging to capture through observational approaches alone. For instance, simulations allow the controlled manipulation of variables enabling the study of “what-if” scenarios that are impractical or unethical to investigate in real-life settings (Fig. 2). Furthermore, these agent-based models could address a key limitation of observational research e.g. by providing a testbed for understanding how internal states (e.g., cognitive processes) interact with external environments over time or by incorporating confounding factors that often bias observational studies.

**Table 1:** Socio-environmental determinants of mental health according to micro-, meso- and macrolevel societal systems

Microlevel	Mesolevel	Macrolevel
Family and Household Dynamics: ❏ Abuse or neglect ❏ Parental mental illness ❏ Conflict or dysfunction Social Relationships: ❏ Bullying or social rejection ❏ Lack of emotional support Early Childhood Adversities: ❏ Trauma and stress	Neighborhood and Community Contexts: ❏ Poverty and deprivation ❏ Crime and violence ❏ Discrimination and social exclusion Access to Social Services: ❏ Lack of mental health services ❏ Unstable housing Workplace and School Environment: ❏ Work stress ❏ Academic pressure	Societal and Cultural Influences: ❏ Economic inequality ❏ Climate change and natural disasters Political and Social Instability: ❏ War and conflict ❏ Global pandemics

**Fig. 3:**
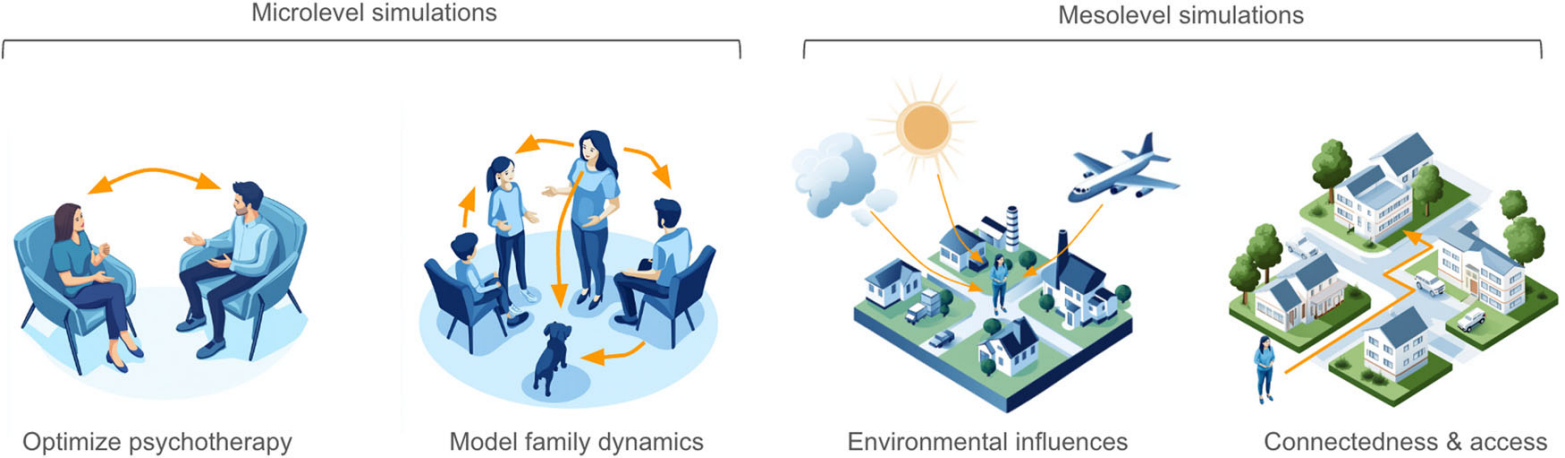
Modern Urban Environments and Their Socio-Environmental Influences on Mental Health. Modern urban environments are characterised by multiple socio-environmental influences and complex micro- and mesolevel interactions with relevance for mental health of inhabitants.

As a specific example, simulations could explore whether adverse events are particularly detrimental during vulnerable developmental periods^51–53^, providing insights into the timing of interventions. Similarly, they could assess how negative social encounters might diminish mental health or how positive interactions could foster resilience and aid in overcoming challenging life circumstances^54,55^. These simulations also provide a novel opportunity to study system-level phenomena, such as how specific family structures might buffer against, or exacerbate, the effects of adversity^56^ or how negative influences could propagate through dysfunctional family systems to indirectly affect others (Fig. 3).

Additionally, agent-based models can simulate the effects of psychotherapeutic interventions on mental health^57,58^, offering a way to tailor treatment strategies to individual needs based on personality structures or other characteristics. By modeling these interventions in-silico, such approaches can optimize resource allocation and refine hypotheses for real-world application. Rather than replacing traditional research methods, these simulations serve as a complementary tool, enhancing our ability to test hypotheses, explore dynamic systems, and bridge gaps in understanding that are otherwise difficult to address. This integrative approach provides actionable insights for clinical practice and policy-making, particularly in tailoring interventions to improve mental health outcomes^40^.

### Meso- and macrolevel simulations of socio-environmental systems

In meso- and macrolevel contexts, factors such as physical properties of the environment or the infrastructure of the community are of increasing importance (Table 1). Environmental stressors like noise, air, light and visual pollution as well as climate change have been shown to have severe effects on mental health and well-being^7,59–61^ which are expected to aggravate in the coming years. Conversely, access to public transportation^26^, green spaces^16^, canals and rivers^62^ as well as sports and healthcare facilities^63^ is essential to support mental health. Using agent-based simulations we can achieve a more detailed understanding of how these factors act and interact to allow a more complete picture of how mental health evolves in dynamic urban environments.

One promising application of agent-based simulations is to explore how combinations of environmental stressors - such as noise, air pollution, and lack of green spaces - affect mental health outcomes in specific urban settings. These simulations could allow researchers to identify which mitigating strategies, such as green space allocation or noise reduction measures, are most effective in reducing the mental health burden. Furthermore, simulations could help to forecast mental health outcomes under hypothetical scenarios, such as rising temperatures or increased urbanization, helping to develop climate-resilient strategies for mental health support.

Another potential application is in guiding urban planning and policy-making. For example, simulations can optimize the placement of parks, healthcare facilities, or public transport stops by modeling their effects on reducing loneliness and improving mental health in underserved areas^64^. They could also enable researchers to explore long-term systemic effects, such as how improved access to public transportation might mitigate the combined impact of environmental and social stressors over longer time periods.

Incorporating additional variables, such as somatic diseases or mobility restrictions, into these simulations provides further insights into vulnerable populations, such as older adults. While aging is often associated with increased health challenges, older adults who remain mentally and physically active can exhibit resilience and improved outcomes, highlighting the potential for active aging. Simulations could assess how urban design improvements, such as increased accessibility, might reduce the mental health burden among ageing populations. Similarly, agent-based models can be used to test interventions aimed at specific demographic groups, allowing for the tailoring of strategies to the needs of diverse communities.

In summary, agent-based simulations serve as a powerful tool for addressing complex research questions that are challenging to tackle through observational or experimental methods alone. By capturing the dynamic interplay between global influences, such as climate change, and locally variable factors, such as air pollution and infrastructure, these models enhance our understanding of the determinants of mental health. They also provide actionable insights for designing evidence-based interventions, informing future public health strategies, and optimizing urban planning to foster mental well-being.

## Validation of Generative Agents in Mental Health Research

Systematic methods need to be developed to validate the accuracy and reliability of generative agents for studying mental health. One promising approach is to replicate established psychological findings within these simulated environments. For instance, the personality trait of neuroticism is consistently associated with a higher risk of affective disorders^65^, and individuals in specific developmental periods (e.g. adolescence) are particularly vulnerable to adverse events that can have lasting effects on mental health^52,66^. To recreate such findings, agents could be assigned developmental characteristics reflective of adolescence, such as heightened emotional sensitivity or increased susceptibility to peer influence, and placed in virtual environments representing real-world contexts like schools, peer groups, and families. Adverse events, such as bullying or social exclusion, can then be introduced as controlled scenarios, enabling researchers to track their impact on the agents over time.

The outcomes of these simulations can be assessed using longitudinal in silico studies. For example, generative agents could periodically complete standardized mental health assessments, such as the PHQ-9 for depressive symptoms^67^, the GAD-7 for anxiety^68^, or tools specifically designed to measure subjective stress levels, such as the Perceived Stress Scale (PSS)^69^ or the Perceived Stress Questionnaire (PSQ)^70^. By measuring changes in these scores over time, researchers can evaluate how adverse experiences during adolescence influence mental health trajectories into adulthood. Comparing these simulated results to empirical findings from longitudinal studies provides a means of validating the models’ ability to mirror real-world psychological phenomena.

Further validation can be achieved by investigating whether the simulated environments can replicate observed empirical patterns or predict novel outcomes. For example, simulations could explore the interaction of neuroticism with environmental stressors, testing whether the predicted outcomes align with real-world data. Additionally, data from digital sensing technologies (e.g., actimetry, speech markers) and ecological momentary assessments can be used to augment agent behaviors, ensuring alignment with real-world behavioral patterns and enhancing the realism of the simulations^71–73^.

Ultimately, the ability of generative agents to consistently replicate both established and novel empirical observations will be critical for their acceptance and utility. Validated models could provide deeper insights into how socio-environmental moderators influence mental health and a powerful tool to explore the mechanisms underlying mental health outcomes and design targeted interventions.

## Challenges in Using Generative Agents for Mental Health Research

While LLMs present significant potential for advancing mental health research by simulating social and environmental determinants, their use is accompanied by several notable challenges. Firstly, most LLMs are designed as general-purpose models which are not specifically tailored to model human behaviour for mental health research. Approaches such as fine-tuning to domain-specific datasets is a common practice that can significantly enhance their performance for targeted use cases including mental health^74^. A related challenge is the limited availability of detailed, high-quality datasets from individuals with validated mental illness diagnoses. This scarcity could hinder the ability to train and validate models that accurately capture the nuances of mental health conditions. One promising source of rich and accurate data to train these models could be audio and video recordings of social interactions, such as normal conversations within families or friends, as well as interactions in psychotherapy sessions. These recordings can capture a wide range of emotional responses and social dynamics, providing detailed and realistic insights into human behavior. Additionally, ecological momentary assessments (EMAs) and audio diaries offer a complementary approach, allowing the collection of emotionally rich data in naturalistic settings with high temporal precision. These methods capture emotional experiences as they occur in daily life, providing an ecologically valid and temporally precise assessment of how emotions evolve in response to real-world contexts.

Another important limitation is that by design LLMs operate within the domain of language. Many facets of mental health risk and resilience operate causally at the origin of individual life (genetic risk) or in early childhood (urban upbringing, many forms of abuse) and it is not immediately obvious how this could be reflected in a language-based agent. Even in adulthood, data show that many resilience factors are influencing behaviour outside awareness (e.g. greenspace visibility in urban contexts^16^) and would not be reflected in language. Even if they were, the question remains how language-based training datasets would reflect such context factors. It is likely that human social behaviours are best represented both in training data and in language based models and might thus be a useful initial focus of using generative agents.

One of the primary ethical concerns involves the inherent biases within LLMs, particularly towards minority groups^75–77^. AI models are often trained on vast datasets that may contain prejudices and stereotypes, which can be inadvertently perpetuated and even amplified when these models are deployed in research or clinical settings^78^. Addressing these biases is crucial to ensure that LLMs do not contribute to discrimination or unequal treatment in mental health interventions. Ethical considerations also include the generalizability of potential findings in mental health derived from generative agents. As the majority of training data for LLMs is derived from western world, it is to be expected that this line of research might not perform well in cultural contexts which are not sufficiently represented in these models. Thus, researchers must consider both minorities underrepresented in training data and vulnerable populations facing systemic disadvantages, such as poverty or severe mental illness.

Furthermore, the capacity of LLMs to model human behaviour presents both opportunities and risks. While these models could potentially be used to promote positive mental health outcomes, there is also the danger of their being exploited to manipulate behaviour in harmful ways. These hazards need to be considered in future research and safeguards need to be established.

Lastly, technical challenges remain with generative agents, particularly the computational demands of simulating large environments like virtual cities, which can cause bottlenecks. Additionally, many mental health researchers lack the programming skills to fully utilize these tools. To address these issues, future efforts should aim to make generative agents more accessible by developing user-friendly platforms and automated processes. This would enable a broader range of researchers to engage with these technologies more effectively, fostering interdisciplinary collaboration.

## Outlook of generative agents in mental health research

Besides the mentioned promising applications of generative agents from mental health research, there is a range of further developments and expansions of this framework which might provide future fruitful research avenues.

As an example, the current form of generative agents includes some form of cognitive architecture consisting of a memory, a retrieval mechanism and the ability to reflect on previous experiences^32^. At the same time, decades of mental health research has generated a wealth of cognitive models ranging from Beck’s cognitive models of depression^79^ or behavioural analysis of stimulus and response^80^ to modern models in the area of computational psychiatry^81–83^. Including these rich models in the context of generative agents might help to stimulate future investigations of the interplay of cognitive processes and socio-environmental influences. Furthermore, there are known biological consequences of exposure to socio-environmental risk factors such as substances of abuse (e.g. cannabis, tobacco), childhood maltreatment, air pollution or loneliness^10,50,84–86^. Expanding generative agents by integrating such biological processes, might improve the modelling of the effects on mental health^27^. Another potential research direction is the investigation of specific policies and how they affect mental health outcomes. As an example Occhipinti et al. employ a system-level approach to simulate how socioeconomic policies can impact suicide rates^87^.

Lastly, generative agents offer a unique platform for fostering interdisciplinary collaboration. Psychologists and psychiatrists can contribute specialised knowledge of mental health, while computer scientists provide expertise in stimulating environments and optimising the use of LLMs. Sociologists and epidemiologists can lend insights into socio-environmental factors, and ethicists can ensure responsible implementation.

## Conclusion

Generative agents powered by LLMs could offer an innovative approach to advancing mental health research by simulating the intricate interplay of socio-environmental determinants on mental health outcomes. By creating realistic virtual environments where agents exhibit human-like behaviours and interactions, researchers could systematically manipulate variables and observe emergent phenomena that are difficult to capture through traditional observational methods. This innovative framework holds the potential to deepen our understanding of mental health dynamics at micro-, meso-, and macro-levels, while also contributing to broader research on whole health, which integrates physical, behavioral and socioeconomic dimensions of well-being.

## Data Availability

No datasets were generated or analysed during the current study.

## References

[1] van den Bosch, M. & Meyer-Lindenberg, A. Environmental exposures and depression: Biological mechanisms and epidemiological evidence. *Annu. Rev. Public Health* **40**, 239–259 (2019). 30633709 10.1146/annurev-publhealth-040218-044106

[2] Solmi, M. et al. Risk and protective factors for personality disorders: An umbrella review of published meta-analyses of case-control and cohort studies. *Front. Psychiatry* **12**, 679379 (2021). 34552513 10.3389/fpsyt.2021.679379 PMC8450571

[3] Alon, N. et al. Social determinants of mental health in major depressive disorder: Umbrella review of 26 meta-analyses and systematic reviews. *Psychiatry Res.* **335**, 115854 (2024). 38554496 10.1016/j.psychres.2024.115854

[4] Arango, C. et al. Risk and protective factors for mental disorders beyond genetics: an evidence-based atlas. *World Psychiatry* **20**, 417–436 (2021). 34505386 10.1002/wps.20894 PMC8429329

[5] Sturm, E. T. et al. Review of major social determinants of health in schizophrenia-spectrum disorders: II. Assessments. *Schizophr. Bull.* **49**, 851–866 (2023). 37022911 10.1093/schbul/sbad024 PMC10318889

[6] Vassos, E., Pedersen, C. B., Murray, R. M., Collier, D. A. & Lewis, C. M. Meta-analysis of the association of urbanicity with schizophrenia. *Schizophr. Bull.* **38**, 1118–1123 (2012). 23015685 10.1093/schbul/sbs096 PMC3494055

[7] Newbury, J. B. et al. Air and noise pollution exposure in early life and mental health from adolescence to young adulthood. *JAMA Netw. Open* **7**, e2412169 (2024). 38805229 10.1001/jamanetworkopen.2024.12169 PMC11134215

[8] Newbury, J. B. et al. Association of air pollution exposure with psychotic experiences during adolescence. *JAMA Psychiatry* **76**, 614–623 (2019). 30916743 10.1001/jamapsychiatry.2019.0056 PMC6499472

[9] Lederbogen, F., Haddad, L. & Meyer-Lindenberg, A. Urban social stress-risk factor for mental disorders. The case of schizophrenia. *Environ. Pollut.* **183**, 2–6 (2013). 23791151 10.1016/j.envpol.2013.05.046

[10] Xu, J. et al. Effects of urban living environments on mental health in adults. *Nat. Med.* **29**, 1456–1467 (2023). 37322117 10.1038/s41591-023-02365-w PMC10287556

[11] Solmi, M. et al. An umbrella review of candidate predictors of response, remission, recovery, and relapse across mental disorders. *Mol. Psychiatry* **28**, 3671–3687 (2023). 37957292 10.1038/s41380-023-02298-3 PMC10730397

[12] Jester, D. J. et al. Review of major social determinants of health in schizophrenia-spectrum psychotic disorders: I. clinical outcomes. *Schizophr. Bull.* **49**, 837–850 (2023). 37022779 10.1093/schbul/sbad023 PMC10318890

[13] Dragioti, E. et al. Global population attributable fraction of potentially modifiable risk factors for mental disorders: a meta-umbrella systematic review. *Mol. Psychiatry* **27**, 3510–3519 (2022). 35484237 10.1038/s41380-022-01586-8 PMC9708560

[14] Gallagher, M. W., Long, L. J. & Phillips, C. A. Hope, optimism, self-efficacy, and posttraumatic stress disorder: A meta-analytic review of the protective effects of positive expectancies. *J. Clin. Psychol.* **76**, 329–355 (2020). 31714617 10.1002/jclp.22882

[15] Ungar, M. & Theron, L. Resilience and mental health: how multisystemic processes contribute to positive outcomes. *Lancet Psychiatry* **7**, 441–448 (2020). 31806473 10.1016/S2215-0366(19)30434-1

[16] Tost, H. et al. Neural correlates of individual differences in affective benefit of real-life urban green space exposure. *Nat. Neurosci.* **22**, 1389–1393 (2019). 31358990 10.1038/s41593-019-0451-y

[17] United Nations: Department of Economic and Social Affairs: Population Division. *World Urbanization Prospects: The 2018 Revision*. (United Nations, New York, NY, 2019).

[18] Marinescu, I. E., Lawlor, P. N. & Kording, K. P. Quasi-experimental causality in neuroscience and behavioural research. *Nat. Hum. Behav.* **2**, 891–898 (2018). 30988445 10.1038/s41562-018-0466-5

[19] Guloksuz, S., van Os, J. & Rutten, B. P. F. The exposome paradigm and the complexities of environmental research in psychiatry. *JAMA Psychiatry* **75**, 985–986 (2018). 29874362 10.1001/jamapsychiatry.2018.1211

[20] Gibbons, R. D. et al. Social vulnerability and prevalence and treatment for mental health and substance use disorders. *JAMA Psychiatry* **81**, 976–984 (2024). 39046728 10.1001/jamapsychiatry.2024.1870 PMC11446668

[21] Tost, H., Champagne, F. A. & Meyer-Lindenberg, A. Environmental influence in the brain, human welfare and mental health. *Nat. Neurosci.* **18**, 1421–1431 (2015). 26404717 10.1038/nn.4108

[22] Stepniak, B. et al. Accumulated environmental risk determining age at schizophrenia onset: a deep phenotyping-based study. *Lancet Psychiatry* **1**, 444–453 (2014). 26361199 10.1016/S2215-0366(14)70379-7

[23] Bonabeau, E. Agent-based modeling: methods and techniques for simulating human systems. *Proc. Natl Acad. Sci. USA* **99**, 7280–7287 (2002). 12011407 10.1073/pnas.082080899 PMC128598

[24] Badham, J. et al. Developing agent-based models of complex health behaviour. *Health Place* **54**, 170–177 (2018). 30290315 10.1016/j.healthplace.2018.08.022 PMC6284360

[25] Silverman, B. G., Hanrahan, N., Bharathy, G., Gordon, K. & Johnson, D. A systems approach to healthcare: agent-based modeling, community mental health, and population well-being. *Artif. Intell. Med.* **63**, 61–71 (2015). 25801593 10.1016/j.artmed.2014.08.006

[26] Yang, Y. et al. Public transit and depression among older adults: using agent-based models to examine plausible impacts of a free bus policy. *J. Epidemiol. Community Health* **74**, 875–881 (2020). 32535549 10.1136/jech-2019-213317

[27] Tracy, M., Cerdá, M. & Keyes, K. M. Agent-based modeling in public health: Current applications and future directions. *Annu. Rev. Public Health* **39**, 77–94 (2018). 29328870 10.1146/annurev-publhealth-040617-014317 PMC5937544

[28] Chao, D., Hashimoto, H. & Kondo, N. Dynamic impact of social stratification and social influence on smoking prevalence by gender: An agent-based model. *Soc. Sci. Med.* **147**, 280–287 (2015). 26610078 10.1016/j.socscimed.2015.08.041

[29] Cherng, S. T., Tam, J., Christine, P. J. & Meza, R. Modeling the effects of E-cigarettes on smoking behavior: Implications for future adult smoking prevalence. *Epidemiology* **27**, 819–826 (2016). 27093020 10.1097/EDE.0000000000000497 PMC5039081

[30] Gorman, D. M., Mezic, J., Mezic, I. & Gruenewald, P. J. Agent-based modeling of drinking behavior: a preliminary model and potential applications to theory and practice. *Am. J. Public Health* **96**, 2055–2060 (2006). 17018835 10.2105/AJPH.2005.063289 PMC1751811

[31] Scott, N. et al. The effects of extended public transport operating hours and venue lockout policies on drinking-related harms in Melbourne, Australia: Results from SimDrink, an agent-based simulation model. *Int. J. Drug Policy* **32**, 44–49 (2016). 27140432 10.1016/j.drugpo.2016.02.016

[32] Park, J. S. et al. Generative agents: Interactive simulacra of human behavior. a*rXiv [cs.HC]* (2023).

[33] Wang, G. et al. Voyager: An open-ended embodied agent with large language models. *arXiv [cs.AI]* (2023).

[34] Chen, J. et al. From persona to personalization: A survey on role-Playing Language Agents. a*rXiv [cs.CL]* (2024).

[35] Grand, G., Blank, I. A., Pereira, F. & Fedorenko, E. Semantic projection recovers rich human knowledge of multiple object features from word embeddings. *Nat. Hum. Behav.* **6**, 975–987 (2022). 35422527 10.1038/s41562-022-01316-8 PMC10349641

[36] Shanahan, M., McDonell, K. & Reynolds, L. Role play with large language models. *Nature* **623**, 493–498 (2023). 37938776 10.1038/s41586-023-06647-8

[37] Ben-Zion, Z. et al. ‘Chat-GPT on the Couch’: Assessing and Alleviating State Anxiety in Large Language Models. preprint https://osf.io/preprints/psyarxiv/j7fwb (2024). 10.1038/s41746-025-01512-6 PMC11876565 40033130

[38] Coda-Forno, J. et al. Inducing anxiety in large language models increases exploration and bias. *arXiv [cs.CL]* (2023).

[39] Binz, M. & Schulz, E. Turning large language models into cognitive models. *arXiv [cs.CL]* (2023).

[40] Volkmer, S., Meyer-Lindenberg, A. & Schwarz, E. Large language models in psychiatry: Opportunities and challenges. *Psychiatry Res.* **339**, 116026 (2024). 38909412 10.1016/j.psychres.2024.116026

[41] Shieh, A. et al. Assessing ChatGPT 4.0’s test performance and clinical diagnostic accuracy on USMLE STEP 2 CK and clinical case reports. *Sci. Rep.* **14**, 9330 (2024). 38654011 10.1038/s41598-024-58760-x PMC11039662

[42] Tu, T. et al. Towards Conversational Diagnostic AI. *arXiv [cs.AI]* (2024).

[43] Kambeitz, J., Schiffman, J., Kambeitz-Ilankovic, L., Ettinger, U. & Vogeley, K. The empirical structure of psychopathology is represented in large language models. preprint https://www.researchsquare.com/article/rs-3347850/v1 (2023).

[44] Betz, L. T., Rosen, M., Salokangas, R. K. R. & Kambeitz, J. Disentangling the impact of childhood abuse and neglect on depressive affect in adulthood: A machine learning approach in a general population sample. *J. Affect. Disord.* **315**, 17–26 (2022). 35882299 10.1016/j.jad.2022.07.042

[45] Haidl, T. K. et al. The non-specific nature of mental health and structural brain outcomes following childhood trauma. *Psychol. Med.* **53**, 1005–1014 (2023). 34225834 10.1017/S0033291721002439

[46] Haidl, T. K. et al. Is there a diagnosis-specific influence of childhood trauma on later educational attainment? A machine learning analysis in a large help-seeking sample. *J. Psychiatr. Res.* **138**, 591–597 (2021). 33992982 10.1016/j.jpsychires.2021.04.040

[47] Singham, T. et al. Concurrent and longitudinal contribution of exposure to bullying in childhood to mental health: The role of vulnerability and resilience. *JAMA Psychiatry* **74**, 1112–1119 (2017). 28979965 10.1001/jamapsychiatry.2017.2678 PMC5710218

[48] Arseneault, L. The long-term impact of bullying victimization on mental health. *World Psychiatry* **16**, 27–28 (2017). 28127927 10.1002/wps.20399 PMC5269482

[49] Beutel, M. E. et al. Loneliness in the general population: prevalence, determinants and relations to mental health. *BMC Psychiatry* **17**, 97 (2017). 28320380 10.1186/s12888-017-1262-x PMC5359916

[50] Benedyk, A. et al. Real-life behavioral and neural circuit markers of physical activity as a compensatory mechanism for social isolation. *Nat. Ment. Health* **2**, 337–342 (2024).

[51] Orben, A., Meier, A., Dalgleish, T. & Blakemore, S.-J. Mechanisms linking social media use to adolescent mental health vulnerability. *Nat. Rev. Psychol.* **3**, 407–423 (2024).

[52] Uhlhaas, P. J. et al. Towards a youth mental health paradigm: a perspective and roadmap. *Mol. Psychiatry* **28**, 3171–3181 (2023). 37580524 10.1038/s41380-023-02202-z PMC10618105

[53] McGorry, P. D. et al. The Lancet Psychiatry Commission on youth mental health. *Lancet Psychiatry* **11**, 731–774 (2024). 39147461 10.1016/S2215-0366(24)00163-9

[54] Monninger, M. et al. Real-time individual benefit from social interactions before and during the lockdown: the crucial role of personality, neurobiology and genes. *Transl. Psychiatry* **12**, 28 (2022). 35064105 10.1038/s41398-022-01799-z PMC8777449

[55] Meyer-Lindenberg, A. & Tost, H. Neural mechanisms of social risk for psychiatric disorders. *Nat. Neurosci.* **15**, 663–668 (2012). 22504349 10.1038/nn.3083

[56] Chen, P. & Harris, K. M. Association of positive family relationships with mental health trajectories from adolescence to midlife. *JAMA Pediatr.* **173**, e193336 (2019). 31589247 10.1001/jamapediatrics.2019.3336 PMC6784807

[57] Hodson, N. & Williamson, S. Can large language models replace therapists? Evaluating performance at simple cognitive behavioral therapy tasks. *JMIR AI* **3**, e52500 (2024). 39078696 10.2196/52500 PMC11322688

[58] Stade, E. C. et al. Large language models could change the future of behavioral healthcare: a proposal for responsible development and evaluation. *Npj Ment. Health Res.* **3**, 12 (2024). 38609507 10.1038/s44184-024-00056-z PMC10987499

[59] Hegewald, J. et al. Traffic noise and mental health: A systematic review and meta-analysis. *Int. J. Environ. Res. Public Health* **17**, 6175 (2020). 32854453 10.3390/ijerph17176175 PMC7503511

[60] Radua, J. et al. Impact of air pollution and climate change on mental health outcomes: an umbrella review of global evidence. *World Psychiatry* **23**, 244–256 (2024). 38727076 10.1002/wps.21219 PMC11083864

[61] Thompson, R. et al. Ambient temperature and mental health: a systematic review and meta-analysis. *Lancet Planet. Health* **7**, e580–e589 (2023). 37437999 10.1016/S2542-5196(23)00104-3

[62] Bergou, N. et al. The mental health benefits of visiting canals and rivers: An ecological momentary assessment study. *PLoS One* **17**, e0271306 (2022). 36044408 10.1371/journal.pone.0271306 PMC9432685

[63] Malinowski, F. et al. Urban distance to mental healthcare units and public transport increases duration of untreated psychosis in first-episode patients. *Int. J. Soc. Psychiatry* **69**, 1938–1948 (2023). 37332226 10.1177/00207640231180825

[64] Lavelle Sachs, A. et al. Connecting through nature: A systematic review of the effectiveness of nature-based social prescribing practices to combat loneliness. *Landsc. Urban Plan.* **248**, 105071 (2024).

[65] Hanke, N. et al. Personality traits differentiate patients with bipolar disorder and healthy controls - A meta-analytic approach. *J. Affect. Disord.* **302**, 401–411 (2022). 35041870 10.1016/j.jad.2022.01.067

[66] Solmi, M. et al. Age at onset of mental disorders worldwide: large-scale meta-analysis of 192 epidemiological studies. *Mol. Psychiatry* **27**, 281–295 (2022). 34079068 10.1038/s41380-021-01161-7 PMC8960395

[67] Kroenke, K., Spitzer, R. L. & Williams, J. B. The PHQ-9: validity of a brief depression severity measure. *J. Gen. Intern. Med.* **16**, 606–613 (2001). 11556941 10.1046/j.1525-1497.2001.016009606.x PMC1495268

[68] Spitzer, R. L., Kroenke, K., Williams, J. B. W. & Löwe, B. A brief measure for assessing generalized anxiety disorder: the GAD-7: The GAD-7. *Arch. Intern. Med.* **166**, 1092–1097 (2006). 16717171 10.1001/archinte.166.10.1092

[69] Cohen, S., Kamarck, T. & Mermelstein, R. A global measure of perceived stress. *J. Health Soc. Behav.* **24**, 385–396 (1983). 6668417

[70] Levenstein, S. et al. Development of the Perceived Stress Questionnaire: a new tool for psychosomatic research. *J. Psychosom. Res.* **37**, 19–32 (1993). 8421257 10.1016/0022-3999(93)90120-5

[71] Lahnakoski, J. M., Eickhoff, S. B., Dukart, J. & Schilbach, L. Naturalizing psychopathology-towards a quantitative real-world psychiatry. *Mol. Psychiatry* **27**, 781–783 (2022). 34667260 10.1038/s41380-021-01322-8 PMC9054666

[72] Wenzel, J. et al. Ecological momentary assessment (EMA) combined with unsupervised machine learning shows sensitivity to identify individuals in potential need for psychiatric assessment. *Eur. Arch. Psychiatry Clin. Neurosci.* **274**, 1639–1649 (2024). 37715784 10.1007/s00406-023-01668-w PMC11422424

[73] Myin-Germeys, I. et al. Experience sampling methodology in mental health research: new insights and technical developments. *World Psychiatry* **17**, 123–132 (2018). 29856567 10.1002/wps.20513 PMC5980621

[74] Ji, S. et al. MentalBERT: Publicly Available Pretrained Language Models for Mental Healthcare. *arXiv [cs.CL]* (2021).

[75] Omiye, J. A., Lester, J. C., Spichak, S., Rotemberg, V. & Daneshjou, R. Large language models propagate race-based medicine. *NPJ Digit. Med.* **6**, 195 (2023). 37864012 10.1038/s41746-023-00939-z PMC10589311

[76] Zack, T. et al. Assessing the potential of GPT-4 to perpetuate racial and gender biases in health care: a model evaluation study. *Lancet Digit. Health* **6**, e12–e22 (2024). 38123252 10.1016/S2589-7500(23)00225-X

[77] Hofmann, V., Kalluri, P. R., Jurafsky, D. & King, S. AI generates covertly racist decisions about people based on their dialect. *Nature* **633**, 147–154 (2024). 39198640 10.1038/s41586-024-07856-5 PMC11374696

[78] Şahin, D. et al. Algorithmic fairness in precision psychiatry: analysis of prediction models in individuals at clinical high risk for psychosis. *Br. J. Psychiatry* **224**, 55–65 (2024). 37936347 10.1192/bjp.2023.141

[79] Beck, A. T. Clinical, Experimental, and Theoretical Aspects. (Hoeber Medical Division, Harper & Row, 1967).

[80] Kanfer, F. H. & Saslow, G. Behavioral analysis: An alternative to diagnostic classification. *Arch. Gen. Psychiatry* **12**, 529–538 (1965). 14286879 10.1001/archpsyc.1965.01720360001001

[81] Hauser, T. U., Skvortsova, V., De Choudhury, M. & Koutsouleris, N. The promise of a model-based psychiatry: building computational models of mental ill health. *Lancet Digit. Health* **4**, e816–e828 (2022). 36229345 10.1016/S2589-7500(22)00152-2 PMC9627546

[82] Friston, K. Computational psychiatry: from synapses to sentience. *Mol. Psychiatry* **28**, 256–268 (2023). 36056173 10.1038/s41380-022-01743-z PMC7614021

[83] Montague, P. R., Dolan, R. J., Friston, K. J. & Dayan, P. Computational psychiatry. *Trends Cogn. Sci.* **16**, 72–80 (2012). 22177032 10.1016/j.tics.2011.11.018 PMC3556822

[84] Penzel, N. et al. Association between age of cannabis initiation and gray matter covariance networks in recent onset psychosis. *Neuropsychopharmacology* **46**, 1484–1493 (2021). 33658653 10.1038/s41386-021-00977-9 PMC8209059

[85] Teicher, M. H., Samson, J. A., Anderson, C. M. & Ohashi, K. The effects of childhood maltreatment on brain structure, function and connectivity. *Nat. Rev. Neurosci.* **17**, 652–666 (2016). 27640984 10.1038/nrn.2016.111

[86] Jeste, D. V. et al. Review of major social determinants of health in schizophrenia-spectrum psychotic disorders: III. *Biol. Schizophr. Bull.* **49**, 867–880 (2023). 10.1093/schbul/sbad031 PMC10318888 37023360

[87] Occhipinti, J.-A. et al. Reducing youth suicide: systems modelling and simulation to guide targeted investments across the determinants. *BMC Med.* **19**, 61 (2021). 33706764 10.1186/s12916-021-01935-4 PMC7952221

